# Oxidative stress in NSC-741909-induced apoptosis of cancer cells

**DOI:** 10.1186/1479-5876-8-37

**Published:** 2010-04-16

**Authors:** Xiaoli Wei, Wei Guo, Shuhong Wu, Li Wang, Peng Huang, Jinsong Liu, Bingliang Fang

**Affiliations:** 1 Department of Biochemical Pharmacology, Beijing Institute of Pharmacology and Toxicology, Beijing 100850, China; 2 Department of Thoracic and Cardiovascular Surgery, The University of Texas MD Anderson Cancer Center, Houston, Texas 77030, USA; 3 Department of Molecular Pathology, The University of Texas MD Anderson Cancer Center, Houston, Texas 77030, USA; 4 Department of Pathology, The University of Texas MD Anderson Cancer Center, Houston, Texas 77030, USA

## Abstract

**Background:**

NSC-741909 is a novel anticancer agent that can effectively suppress the growth of several cell lines derived from lung, colon, breast, ovarian, and kidney cancers. We recently showed that NSC-741909-induced antitumor activity is associated with sustained Jun N-terminal kinase (JNK) activation, resulting from suppression of JNK dephosphorylation associated with decreased protein levels of MAPK phosphatase-1. However, the mechanisms of NSC-741909-induced antitumor activity remain unclear. Because JNK is frequently activated by oxidative stress in cells, we hypothesized that reactive oxygen species (ROS) may be involved in the suppression of JNK dephosphorylation and the cytotoxicity of NSC-741909.

**Methods:**

The generation of ROS was measured by using the cell-permeable nonfluorescent compound H_2_DCF-DA and flow cytometry analysis. Cell viability was determined by sulforhodamine B assay. Western blot analysis, immunofluorescent staining and flow cytometry assays were used to determine apoptosis and molecular changes induced by NSC-741909.

**Results:**

Treatment with NSC-741909 induced robust ROS generation and marked MAPK phosphatase-1 and -7 clustering in NSC-741909-sensitive, but not resistant cell lines, in a dose- and time-dependent manner. The generation of ROS was detectable as early as 30 min and ROS levels were as high as 6- to 8-fold above basal levels after treatment. Moreover, the NSC-741909-induced ROS generation could be blocked by pretreatment with antioxidants, such as nordihydroguaiaretic acid, aesculetin, baicalein, and caffeic acid, which in turn, inhibited the NSC-741909-induced JNK activation and apoptosis.

**Conclusion:**

Our results demonstrate that the increased ROS production was associated with NSC-741909-induced antitumor activity and that ROS generation and subsequent JNK activation is one of the primary mechanisms of NSC-741909-mediated antitumor cell activity.

## Background

We recently identified a small molecule (oncrasin-1) through cell-based synthetic lethality screening that can effectively kill several lung cancer cell lines harboring mutant K-Ras genes [1]. Subsequent analyses of oncrasin-1 analogues led us to identify several active compounds with similar chemical structures. NSC-741909 is one of the oncrasin-1 analogues that was highly active against several cell lines derived from lung, colon, breast, ovarian, and kidney cancers when tested in NCI-60 cancer cell lines by the Developmental Therapeutics Program at the National Cancer Institute. Using a reverse-phase protein microarray assay, we determined molecular changes in 77 protein biomarkers in an oncrasin-sensitive lung cancer cell line after treatment with NSC-741909 [2]. These results showed that treatment with NSC-741909 induced persistent activation of mitogen-activated protein kinases (MAPKs), including p38 MAPK, Jun N-terminal kinase (JNK), and extracellular signal-regulated kinase (ERK), and that persistent JNK activation is associated with apoptosis induction by this compound [2]. Further studies revealed that treatment with NSC-741909 suppressed MAPK phosphatase-1 expression and JNK dephosphorylation, in a dose-dependent manner [2]. Those results suggest that inhibition of JNK dephosphorylation is one of the molecular mechanisms critical for the NSC-741909-induced sustained activation of JNK and cell death.

JNKs are activated by dual phosphorylation on the Thr-Pro-Tyr motif in the activation loop through mitogen-activated protein kinase kinase 4 (MKK4) and 7 (MKK7) and inactivated by dephosphorylation through a group of MAP kinase phosphatases [3]. MAP kinase phosphatases (MKPs) are a group of dual-specificity phosphatases that inactivate MAPKs by dephosphorylating their threonine and tyrosine residues. At least 16 mammalian dual-specificity phosphatases that can dephosphorylate MAPKs have been identified [4]. Their tissue-specific transcriptional regulation, expression patterns, substrate specificities, and subcellular localization play critical roles in controlling MAPK activity and signal transduction in each cell type [4]. Accumulating evidence has demonstrated that, like other protein tyrosine phosphatases, the conserved catalytic cysteine residue in the active motif of MKPs is highly susceptible to reversible oxidation by local reactive oxygen species (ROS) such as hydrogen peroxide (H_2_O_2_) [5,6], which leads to inactivation of MKPs and activation of MAPKs [7-9]. ROS-mediated inhibition of MKPs is critical for TNFα-induced sustained activation of JNK and subsequent apoptosis [7]. Interestingly, ROS were recently identified as common mediators of antibiotic-induced cell death in bacteria [10]. Moreover, many anticancer drugs act as prooxidants, which may trigger the generation of free radicals, such as ROS or reactive nitrogen species [11,12], and promote apoptosis. In fact, ROS-induced oxidative stress and cell death play important roles in the efficacy of many antineoplastic agents [13,14].

To investigate whether oxidative stress is involved in the cytotoxicity of oncrasin compounds, we examined the production of ROS and its effects on JNK activation and cell death after treatment of oncrasin-sensitive and -resistant cells with NSC-741909. We found that ROS formation is an important component of NSC-741909-induced apoptosis. Furthermore, the NSC-741909-induced generation of ROS, cytotoxicity, and JNK activation, could be dramatically attenuated by some antioxidants, such as nordihydroguaiaretic acid, aesculetin, baicalein, and caffeic acid.

## Methods

### Cell lines and cell culture conditions

The human non-small cell lung carcinoma cell lines H460, H157, H322, and H1299 were grown in Dulbecco's modified Eagle's medium supplemented with 10% fetal bovine serum and 100 mg/mL penicillin-streptomycin (all from Life Technologies, Gaithersburg, MD, USA). Normal bronchial epithelial cells (HBEC) were kindly provided by Dr. John Minna (Southwest Medical School, Dallas, TX) and were cultured in serum-free keratinocyte medium (Invitrogen Corporation, Carlsbad, CA). Cells were cultured at 37°C in a humidified incubator containing 5% CO_2_.

### Chemicals and antibodies

NSC-741909 (the structure was shown in additional file 1) was synthesized by Zhejiang Yuancheng MST Inc. (Hangzhou, China). This compound was 98.5% pure, as determined by high-performance liquid chromatography--mass spectrometry (LC/MS) analysis. The chemical structure was confirmed by nuclear magnetic resonance. N-acetylcysteine (NAC), rotenone, Nω-nitro-L-arginine methyl ester (L-NAME), diallyl sulfide (DSE), naproxen, oxypurinol, nordihydroguaiaretic acid (NDGA), baicalein, caffeic acid, MK886, and zileuton were purchased from Calbiochem (San Diego, CA, USA). Antibodies to the following proteins were used for Western blot analysis: JNK, phospho-JNK, phospho-c-Jun (Cell Signaling Technology, Danvers, MA, USA), poly-(ADP-ribose) polymerase (BD Biosciences Pharmingen, San Diego, CA, USA), MKP1 (Santa Crutz, CA, USA), MKP7 (Sigma-Aldrich, St. Louis, MO, USA), caspase-8 (Alexis Biochemicals, Farmingdale, NY, USA), β-actin, and hemagglutinin (HA) (Sigma-Aldrich, St. Louis, MO, USA). 2',7'-Dichlorofluorescein diacetate (H_2_DCF-DA) was purchased from Invitrogen Molecular Probes (Carlsbad, CA, USA).

### ROS analysis

The cell-permeable nonfluorescent compound H_2_DCF-DA was used for measuring intracellular ROS. Inside cells, H_2_DCF-DA is de-esterified to 2', 7'-dichlorofluorescein (H_2_DCF), which is further oxidized by ROS to fluorescent dichlorofluorescein (DCF) that remains inside the cells and can be quantified by flow cytometry, as described in the manufacturer's instructions. H_2_DCF-DA was dissolved in dimethylsulfoxide and diluted with phosphate-buffered saline (PBS) to a final concentration of 5 μmol/L. Cells were seeded at a density of 2.5 × 10^5 ^cells/well in six-well plates and allowed to grow overnight. The cells were treated either with different concentrations of NSC-741909 for 6 h or with 1 μM NSC-741909 for different time periods (0.5, 2, 4, 6 h). Subsequently, 5 μmol/L H_2_DCF-DA was added, and cells were incubated for 40 min at 37°C; cells were then returned to a prewarmed growth medium and incubated for 10 min at 37°C. Cells were harvested with trypsin and washed once with PBS, and the fluorescence intensity was determined using flow cytometry, with excitation and emission settings of 488 nm and 530 nm, respectively. The mean fluorescence peak was analyzed from the gated cell population of 10,000 cells. For the NSC-741909-antioxidant combination test, the antioxidants were added 30 min before NSC-741909. All experiments were performed three times. The flow cytometry assays were performed at the Flow Cytometry and Cellular Imaging Facility at The University of Texas M. D. Anderson Cancer Center.

### Cell viability assay

Cells were seeded at a density of 1 × 10^4 ^cells/well in 96-well plates. After overnight incubation, the cells were treated with NSC-741909 (0.03 - 10 μM), either alone or in combination with different antioxidant compounds for 24 h. The antioxidants were added 30 min before NSC-741909 was added, and the inhibitory effects of NSC-741909, alone or in combination with the antioxidants, on cell growth were determined using the sulforhodamine B (SRB) assay, as described previously [15]. We determined the relative cell viability by normalizing the cells to the dimethylsulfoxide-treated control cells, which was set at 100%. Each experiment was performed in quadruplicate and repeated for a total of at least three times.

### Apoptosis analysis

The flow cytometry assay was performed as described previously [16]. In brief, cells were seeded at a density of 2.5 × 10^5 ^cells/well in six-well plates and allowed to grow overnight. The cells were treated with NSC-741909 (1 μM) alone or in combination with different antioxidants for 24 h. The antioxidants were added to the cells 30 min before NSC-741909 was added. After treatment, the cells were harvested with trypsin, washed once with PBS, and fixed by incubation with 70% ethanol overnight at 4°C. Before flow cytometry analysis, cells were stained with propidium iodide (PI; 1 ml PI, 10 μl RNase, 9 ml PBS; final PI concentration of 50 μg/ml) for 30 min. A flow cytometry assay was used to measure the sub-G0/G1 cellular DNA content using Cell Quest software (Becton-Dickinson (Franklin Lakes, NJ, USA). All experiments were performed three times. The flow cytometry assays were performed in the Flow Cytometry and Cellular Imaging Facility at M. D. Anderson Cancer Center.

### Western blot analysis

Cells were washed with cold PBS and subjected to lysis in Laemmli's lysis buffer. The protein concentration was determined using the Bradford method. Equal amounts of lysate (40 μg) were separated by 10% sodium dodecyl sulfate-polyacrylamide gel electrophoresis and then transferred to Hybond-enhanced chemiluminescence membranes (GE Healthcare Life Sciences, Piscataway, NJ, USA). Membranes were then blocked with PBS containing 5% low-fat milk and 0.05% Tween (PBST) for 1 h and then incubated with primary antibodies overnight at 4°C. After being washed three times with PBST, membranes were incubated with peroxidase-conjugated secondary antibodies for 1 h at room temperature. The membranes were washed with PBST again and developed with a chemiluminescence detection kit (ECL kit; GE Healthcare Life Sciences). β-Actin was used as a loading control.

### Immunofluorescent staining

Cells were seeded at a density of 1 × 10^5 ^cells per well in 6-well plates containing a 1% gelatin-treated cover slide. Cells were allowed to grow overnight. Cells were treated with 1 μM NSC-741909 for different time periods as indicated. After the treatment, cells were washed with PBS twice, then fixed with 2% paraformaldehyde for 20 min, permeablized with 0.1% Triton-100 for 20 min, and blocked with 5% normal goat serum for 1 h. The slides were incubated with primary antibodies followed by FITC or Rhodamine-linked secondary antibodies. After washed with PBS thrice, the slides were taken out and mounted with Prolong Gold antifade reagent (Molecular Probes, Carsbad, CA, USA). The slides were read under Olympus fluorescence microscope (Olympus, Melville, NY, USA).

### Statistical analysis

Statistical differences between treatment groups were assessed by analysis of variance (ANOVA) using StatSoft software (Tulsa, OK, USA). P values of < 0.05 were regarded as significant.

## Results

### NSC-741909 induced MKP1 and MKP7 clustering and generation of reactive oxygen species in oncrasin-sensitive cells

**Figure 1:**
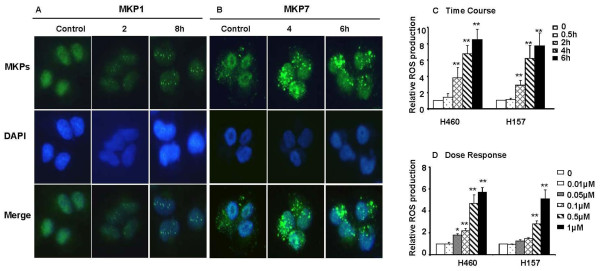
**NSC-741909-induced MKP1 and MKP7 clustering, and ROS production in sensitive cell lines**. (A, B), Clustering of MKP1 (A) and MKP7 (B). H460 cells were treated with 1 μM NSC-741909 for the indicated time periods. MKP1 and MKP7 were detected by immunohistochemical staining. (C) and (D) ROS induction in H460 and H157 cells after treatment with 1 μM NSC-741909 for the indicated time periods (C), or with different concentrations of NSC-741909 (0.01 - 1 μM) for 6 h (D). Cells were stained with 2', 7'-dichlorofluorescein diacetate and the fluorescent cell population was counted by flow cytometry. Cells treated with solvent alone (dimethylsulfoxide, DMSO) were used as controls, and their mean fluorescence intensity was set at 1. Each data point represents the mean ± SD of three independent experiments. *p < 0.05, **p < 0.01, compared with cells treated with DMSO alone.

Our recent study showed that NSC-741909 induced sustained JNK activation associated with decreased protein levels of MKP1, one of MAP kinase phosphatases that inactivate JNK and p38 MAP kinases [2]. Interestingly, NSC-741909 induced an increase of MKP1 mRNA expression in both time- and dose-dependent manner. The peak occurred at 1 h post-treatment, which had 5-10 fold increase when compared with DMSO treated control [2], suggesting that NSC-741909 may suppress MKP1 expression at the post-transcriptional level and that increased MKP1 mRNA expression might reflect a negative feedback to the decrease of its protein levels. Because MKPs are highly susceptible to oxidative stress, which can induce aggregations of MKPs, we further tested MKP1 and MKP7 statuses by immunohistochemical staining after treatment with NSC-741909. The result showed that treatment of H460 cells with 1 μM of NSC-741909 induced cluster formation of MKP1 and MKP7 at all time points examined (2 - 8 h after the treatment) (Fig. 1A, B), suggesting that oxidative stress might play roles in alteration of MKP1 and MKP7, both are responsible for inactivating JNK through dephosphorylation.

To determine whether treatment with NSC-741909 would generate oxidative stress in sensitive cells, we treated two sensitive lung cancer cell lines, H460 and H157, with 1 μM NSC-741909. Cells were stained with H_2_DCF-DA, and were examined for the production of ROS by measuring the cell population with positive DCF-derived fluorescence at various time points after the NSC-741909 treatment. Cells treated with solvent alone (dimethylsulfoxide) and stained with H_2_DCF-DA were used as controls. We found that treatment with NSC-741909 stimulated ROS generation in a time-dependent manner in both cell lines, in contrast to the control cells (Fig. 1C). An increase in the amount of ROS generated occurred as early as 30 min to 1 h after treatment and was as high as 6- to 8-fold above baseline levels after 6 h. Similar results were obtained with cells that were treated with the lead compound, oncrasin-1 (data not shown). We then evaluated the generation of ROS as a function of NSC-741909 concentration 6 h after treatment with NSC-741909. The result showed that the generation of ROS by NSC-741909 was dose-dependent and detectable at a dose of 50 nM in both cell lines (Fig. 1D).

### Association between NSC-741909-induced generation of reactive oxygen species and suppression of cell growth

**Figure 2:**
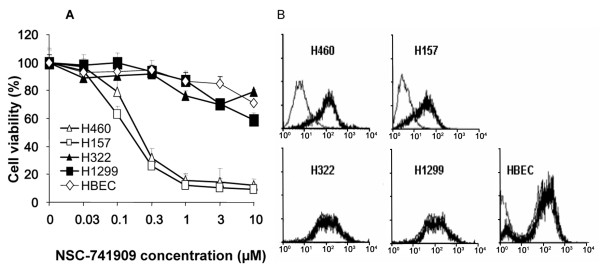
**Antitumor cell activity of NSC-741909 is associated with ROS generation**. (A) Two sensitive (H460 and H157 cells) and two resistant lung cancer cell lines (H322 and H1299) were treated with different concentrations of NSC-741909 (0.03 - 10 μM). Cell viability was determined 24 h after treatment. Cells treated with solvent (dimethylsulfoxide) alone were used as controls, and their viability was set to 100%. Each data point represents the mean ± SD of three independent experiments. (B) The five cell lines were treated with 1 μM NSC-741909 for 6 h and then stained with 2', 7'-dichlorofluorescein diacetate. Fluorescence intensity in cell samples was determined by flow cytometry analysis. Shown here are representative FACS graphs, which show the shift in the fluorescent cell population after NSC-741909 treatment (dark lines) when compared with control cells (light lines).

To investigate whether NSC-741909-mediated ROS generation was correlated with NSC-741909-mediated cell growth suppression, we measured cell viability and ROS generation after NSC-741909 treatment in two sensitive (H460 and H157) and two resistant (H322, H1299) lung cancer cell lines. Normal bronchial epithelial cells (HBEC), which is resistant to NSC-741909, were also included in the studies. The cells were treated with 0.03 - 10 μM NSC-741909 for 24 h and then cell viability was determined by using the SRB assay. To test for the generation of ROS, the cells were treated with 1 μM NSC-741909 for 6 h and then stained with H_2_DCF-DA, as described earlier. The results showed that treatment with NSC-741909 markedly suppressed cell growth in a dose-dependent manner in both the H460 and H157 cells, with a 50% growth-inhibitory concentration of 0.2 μM and 0.1 μM respectively. In comparison, H322, H1299 and HBEC were resistant to NSC-741909, with a 50% growth-inhibitory concentration of more than 10 μM, the highest concentration tested (Fig. 2A). The NSC-741909-induced ROS production paralleled the results of the cell viability experiment; ROS generation increased markedly after exposure of H460 and H157 cells to NSC-741909 (1 μM) for 6 h as compared with the solvent-treated controls (data not shown). In contrast, we did not detect any ROS in H322 and H1299 cells 6 h after NSC-741909 treatment, even at a concentration of 10 μM, although a mild ROS increase (<0.6 fold) was observed in HBEC under the same treatment (Fig. 2B). These data show that the increased ROS production coincides with the suppression of cell growth after NSC-741909 treatment.

### Antioxidant blocks NSC-741909-induced ROS production and suppression of cell growth

**Figure 3:**
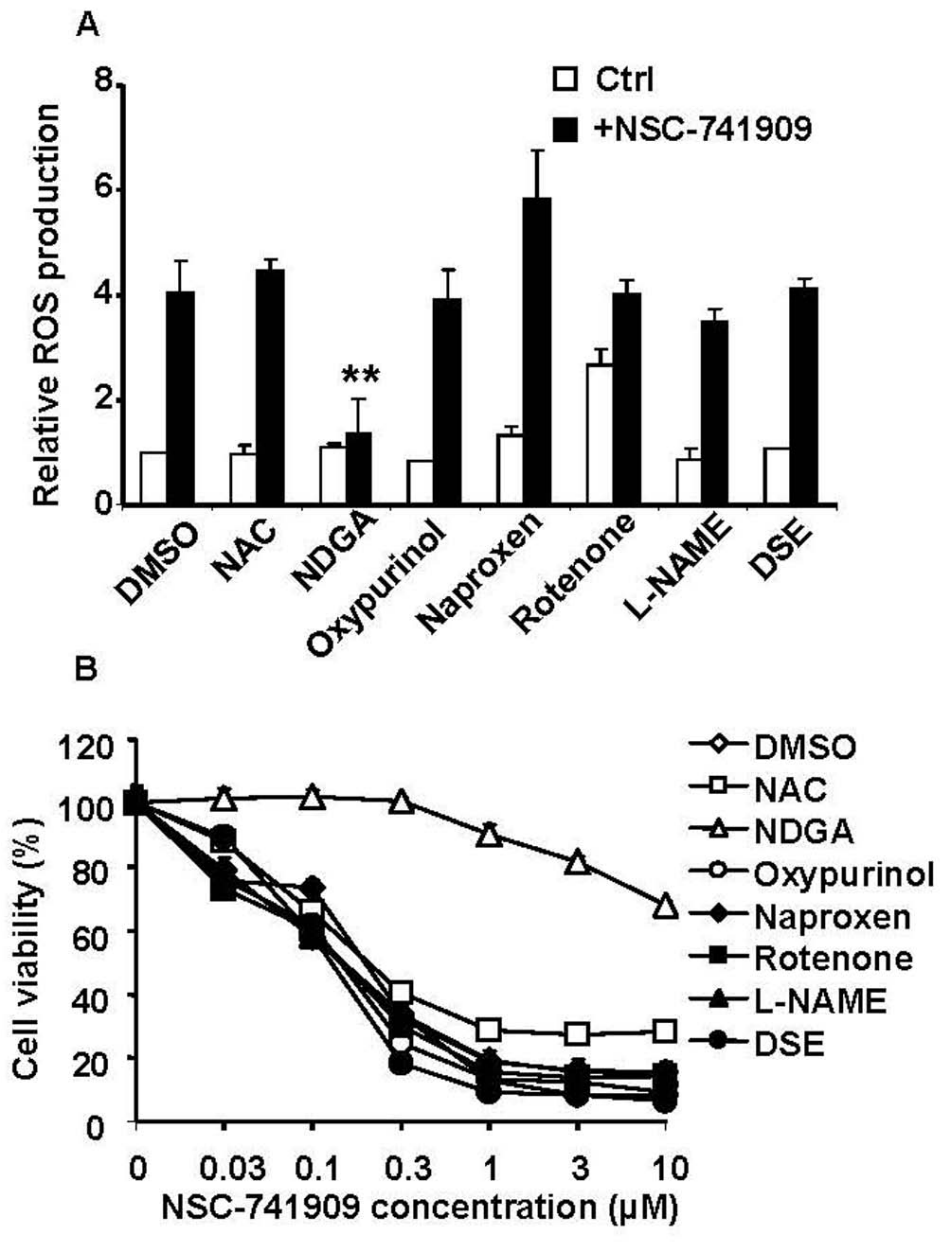
**Effects of antioxidants on ROS production and cell growth suppression induced by NSC-741909**. Cells were treated with 1 μM NSC-741909 for 6 h (for ROS generation) or 24 h (for cell viability) in the presence or absence of different inhibitors. (A) After treatment, cells were stained with 2', 7'-dichlorofluorescein diacetate, and the fluorescent cell population was counted by flow cytometry and the relative ROS production was calculated. **p < 0.01, compared with cells treated with NSC-741909 alone. (B) Cell viability was determined using the sulforhodamine B assay. Cells treated with solvent (dimethylsulfoxide) alone were used as controls, with their viability set at 100%. Each data point represents the mean ± SD of three independent experiments. NAC, N-acetylcysteine; L-NAME, Nω-nitro-L-arginine methyl ester, a nitric-oxide synthase inhibitor; DSE, diallyl sulfide, an inhibitor of cytochrome P450 2E1; naproxen, cyclooxygenase inhibitor; oxypurinol, hypoxanthine/xanthine oxidase inhibitor; and NDGA, nordihydroguaiaretic acid, a lipoxygenase inhibitor.

ROS, such as hydrogen peroxide (H_2_O_2_), superoxide (O^2-^), and hydroxyl radical (OH·), are generated in cells by several pathways. Most cellular O^2- ^is generated during electron transport through the mitochondrial respiratory chain reactions mediated by the coenzyme Q and ubiquinone complexes. O^2- ^is also generated by NADPH cytochrome P450 reductase, hypoxanthine/xanthine oxidase, NADPH oxidase, lipoxygenase (LOX), and cyclooxygenase [17]. Superoxide dismutase converts O^2- ^into H_2_O_2_, and H_2_O_2 _is mostly converted into H_2_O by glutathione (GSH) peroxidase and catalase. H_2_O_2 _produces the highly reactive OH· by the Fenton/Haber-Weiss reaction in the presence of iron [17]. To further examine the role of the ROS generated by treatment of cells with NSC-741909, we evaluated whether the NSC-741909-generated ROS could be inhibited by various antioxidant agents. For this purpose, we treated cells with 10 mM NAC (an antioxidant [18]), 1 μM rotenone (a mitochondrial electron transport chain inhibitor [19]), 300 μM L-NAME (a nitric-oxide synthase inhibitor [20]), 10 μM DSE (an inhibitor of cytochrome P450 2E1 [21]), 300 μM naproxen (a cyclooxygenase inhibitor [22]), 1 mM oxypurinol (a hypoxanthine/xanthine oxidase inhibitor [23]), or 20 μM NDGA (an antioxidant and LOX inhibitor [24]) 30 min prior to the addition of NSC-741909. Generation of ROS was then measured 6 h after treatment with NSC-741909. The results showed that the NSC-741909-induced generation of ROS in H460 cells was substantially diminished by pretreatment with NDGA, but not by pretreatment with any of the other antioxidant agents (Fig. 3A). The cell viability analysis also revealed that only NDGA blocked the NSC-741909-induced growth suppression, with a 50% growth-inhibitory concentration of more than 10-fold shift, whereas NAC, rotenone, L-NAME, DSE, naproxen, and oxypurinol had no obvious effect on cell growth (Fig. 3B). These results indicate that NSC-741909 may induce oxidative stress through a specific pathway that is affected by NDGA or that NDGA is potent antioxidant that can effectively block NSC-741909 induced oxidative stress.

### NDGA inhibits NSC-741909-induced apoptosis

Our previous studies have demonstrated that the reduction in cell viability caused by oncrasin compounds is mainly caused by apoptosis induction [2]. To further evaluate whether NDGA blocks NSC-741909-mediated cell killing, we tested the effects of NDGA and NAC on apoptosis induction by NSC-741909. H460 cells were treated with 1 μM NSC-741909 for 24 h, with or without the prior addition of NDGA or NAC, and the percentage of apoptotic cells was determined by flow cytometry analysis.

**Figure 4:**
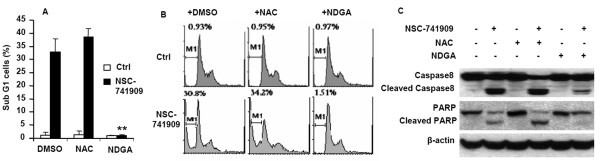
**NDGA inhibits NSC-741909-induced apoptosis and caspase-8 and poly-(ADP-ribose) polymerase (PARP) activation**. H460 cells were treated with 1 μM NSC-741909 in the presence or absence of 10 mM N-acetyl cysteine (NAC) or 20 μM nordihydroguaiaretic acid (NDGA). Cells treated with solvent (dimethylsulfoxide) or the antioxidants alone were used as controls. (A) Percentage of apoptotic cells was determined by flow cytometry analysis 24 h after treatment. The values shown represent the mean ± SD of three analyses. **p < 0.01, compared with cells treated with NSC-741909 alone. (B) Representative FACS graphs. (C) Whole-cell lysates from H460 cells treated as described above were harvested for Western blot analysis of caspase-8 and PARP activation.

The results showed that treatment with NSC-741909 alone led to a dramatic increase in the number of apoptotic cells (those in the sub-G1 phase) as compared with cells treated with solvent (Fig. 4A). Treatment of cells with either NAC (10 mM) or NDGA (20 μM) alone did not affect the cell growth cycle or induce apoptosis. Pretreatment of cells with NDGA (20 μM) markedly decreased the percentage of apoptotic cells compared with NSC-741909 treatment alone (1.7% vs. 32.7%, respectively). In contrast, pretreatment with NAC had no effect on the NSC-741909-induced apoptosis (Fig. 4A and 4B).

The effect of NDGA on NSC-741909-induced apoptosis was further verified by Western blot analysis. Pretreatment of cells with NDGA (20 μM) markedly blocked the NSC-741909-induced activation of caspase-8 and cleavage of poly-(ADP-ribose) polymerase (Fig. 4C). However, pretreatment with NAC did not have a similar effect. Together, these results indicate that NDGA inhibits NSC-741909-mediated apoptosis.

### Effects of other antioxidants on NSC-741909-induced generation of ROS

**Figure 5:**
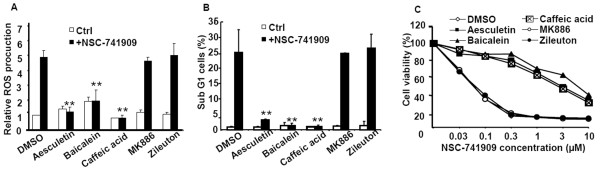
**Effects of other lipoxygenase (LOX) inhibitors on ROS generation and apoptosis induced by NSC-741909**. Cells were treated with 1 μM NSC-741909 for 6 h (for ROS generation) or 24 h (for analysis of apoptosis and cell viability) in the presence or absence of LOX inhibitors. (A) After treatment, cells were stained with 2', 7'-dichlorofluorescein diacetate, and the fluorescent cell population was counted by flow cytometry and relative ROS production was calculated. (B) Percentage of apoptotic cells determined by flow cytometry. **p < 0.01, compared with cells treated with NSC-741909 alone. (C) Percentage of viable cells determined by the sulforhodamine B assay. Cells treated with solvent (dimethylsulfoxide) alone were used as a control, with viability set at 100%. Each data point represents the mean ± SD of three independent experiments.

NDGA is known as an antioxidant and a nonselective LOX inhibitor [25]. In mammalian cells, there are three subtypes of LOX, 5-, 12-, and 15-LOX [26,27]. To investigate whether other LOX inhibitors have effects similar to those of NDGA on NSC-741909-mediated cell death, we evaluated the effects on NSC-741909's antitumor cell activity of several LOX inhibitors, including aesculetin (a nonselective LOX inhibitor), MK886 (an inhibitor of the 5-LOX-activating protein), zileuton (a 5-LOX inhibitor), baicalein (a 12/15-LOX inhibitor), and caffeic acid (a 5/15-LOX inhibitor). The results showed that antioxidants aesculetin (20 μM), baicalein (10 μM), and caffeic acid (10 μM) significantly blocked the NSC-741909-induced ROS generation (Fig. 5A, P < 0.01) and apoptosis (Fig. 5B, P < 0.01), whereas non-antioxidants MK886 (10 μM) and zileuton (20 μM) had no such inhibitory effect. The cell viability analysis further revealed that antioxidants aesculetin (20 μM), baicalein (10 μM), and caffeic acid (10 μM) also significantly reversed the NSC-741909-induced growth suppression at doses of 0.03 - 10 μM, each with a 50% growth-inhibitory concentration of more than 10-fold shift, whereas MK886 (10 μM) and zileuton (20 μM) had no obvious effect on the growth suppression at those concentrations (Fig. 5C). These data suggest that NDGA, aesculetin, baicalein and caffeic acid may block NSC-741909-induced ROS independent of Lox activities. In fact, siRNA of 5-, 12-, and 15-Lox had no effects on NSC-741909-induced ROS generation and cell killing (Additional file 2), further indicating that those antioxidants mediated antagonist effect was independent of Lox activities.

### Suppression of NSC-741909-induced JNK/c-Jun activation by antioxidants

**Figure 6:**
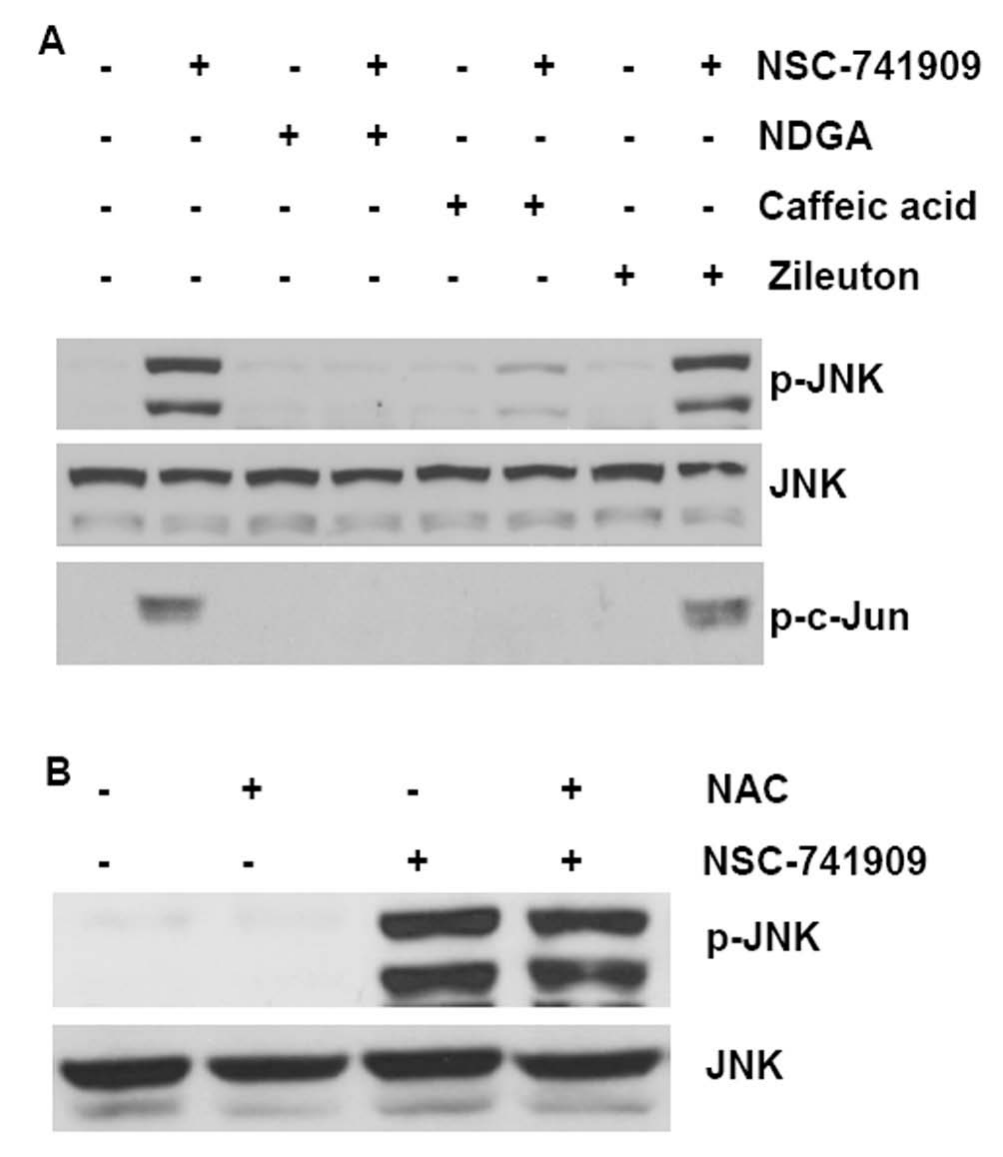
**Effects of antioxidants on NSC-741909-induced JNK/c-Jun activation**. **(A) **H460 cells were treated with 1 μM NSC-741909 in the presence or absence of various lipoxygenase inhibitors for 24 h. **(B) **H460 cells were treated with 1 μM NSC-741909 in the presence or absence of 10 mM of N-acetylcysteine (NAC) for 24 h. Whole-cell lysates were harvested for Western blot analysis of JNK and c-Jun activation.

We recently found that sustained activation of JNK contributes to the apoptosis induced by NSC-741909 [2]. Therefore, we tested whether the antioxidants that can block the NSC-741909-induced generation of ROS and apoptosis can also inhibit the NSC-741909-induced activation of JNK and its downstream target c-Jun. We found that treatment of H460 cells with either NDGA (20 μM) or caffeic acid (10 μM) alone had no effect on the expression of either JNK or c-Jun, but pretreatment of cells with NDGA (20 μM) or caffeic acid (10 μM) markedly blocked the NSC-741909-induced phosphorylation of JNK and c-Jun, without any obvious effect on the basal JNK level (Fig. 6A). These data showed that either NDGA (20 μM) or caffeic acid (10 μM) was sufficient to block the NSC-741909-mediated activation of JNK. In comparison, zileuton, which had no effect on NSC-741909-induced ROS generation and apoptosis induction, also had no effect on the phosphorylation of JNK and c-Jun induced by NSC-741909 (Figures 6A and 6B). These data further indicate that NSC-741909-induced generation of ROS may contribute to the sustained activation of JNK in oncrasin-sensitive cells, which in turn, is critical for NSC-741909-mediated apoptosis.

## Discussion

Our results demonstrate that NSC-741909 can effectively induce ROS generation in oncrasin-sensitive, but not in the resistant human lung cancer cells. Blocking NSC-741909-induced ROS generation with some antioxidants, such as NDGA, aesculetin, baicalein, and caffeic acid, effectively blocked NSC-741909-induced cell death. Furthermore, these antioxidants also blocked JNK activation, demonstrating that ROS generation is one of the primary mechanisms by which NSC-741909 induces the sustained activation of JNK and apoptosis.

ROS are constantly generated and eliminated inside a cell. The balance of ROS can be dramatically affected by many environmental stimuli, including cytokines, growth factors, ultraviolet radiation, radiotherapy, and chemotherapeutic agents. ROS generation and subsequent oxidative damage to the cell membrane is one of the major mechanisms of radiotherapy-mediated apoptotic cell death [28]. Similarly, many chemotherapeutic agents, including cisplatin [29], paclitaxel [30], doxorubicin [31,32], and the histone deacetylase inhibitor suberoylanilide hydroxamic acid, induce ROS generation in target cells [33]. Moreover, scavenging of ROS with antioxidants causes cells to resist apoptosis induced by gamma-irradiation and various chemotherapeutic agents [34].

Interestingly, ROS production is often elevated in oncogene-transformed cells. For example, transformation of cells with oncogenic Ras leads to increased production of O^2-^, which can be suppressed by the expression of dominant-negative isoforms of Ras or Rac1 [35,36]. Similarly, increased Akt activity sensitizes cells to ROS-mediated apoptosis by increasing the intracellular concentration of ROS through increased oxygen consumption and inhibition of the expression of ROS scavengers downstream of FoxO, particularly manganese superoxide dismutase [37] and sestrin 3 [38]. In addition, overexpression of growth factor receptors, such as insulin growth factor receptor [39], epidermal growth factor receptor [40], and vascular endothelial growth factor receptor [41], leads to increased generation of ROS. Those changes were frequently found in malignant cells.

Elevated levels of ROS in oncogene-transformed or tumor cells potentiate the oxidative stress-mediated activation of MAP kinases, particularly the JNK and p38 kinases, which sensitizes those cells to chemotherapeutic drug- and radiation-induced cell death [42]. Thus, oxidative stress associated with increased activities of oncogenes and growth factor receptors represents a specific vulnerability of malignant cancer cells that can be selectively targeted by novel oxidative stress-inducing anticancer agents such as NSC-741909. It has been well documented that MAPKs, such as JNK, are redox sensitive and involved in apoptosis signaling [12,43,44]. There are two mechanisms of JNK activation: the earlier and transient activation occurs through the pro-inflammatory cytokine signaling cascade, and the delayed and sustained activation is mediated by ROS [45], which inactivate MAP kinase phosphatases by reacting with catalytic cysteine and causing their aggregation. Our results also showed that treatment with NSC-741909 induced clustering of MKP1 and MKP7 in sensitive cells, suggesting that the ROS-mediated inactivation of MKPs is a primary mechanism by which NSC-741909 activates JNK signaling pathway and exerts its antitumor cell effect. However, cellular levels of MKPs are likely not the critical factors for the sensitivity to NSC-741909 in lung cancer cells, because levels of MKPs in microarrays for lung cancer cell lines described here and that in the NCI's 60 cell line panel did not reveal obvious association between IC_50_s and MKP expression levels (Additional file 3). This may be explained by the fact that MKPs are down stream of ROS in inactivating JNK. Factors that directly contribute to ROS inductions might be more important for apoptosis induction by NSC-741909. Nevertheless, the underlying mechanisms or the sources of NSC-741909 induced ROS remain to be characterized.

Our results showed several antioxidants, including NDGA, aesculetin, baicalein, and caffeic acid, can block NSC-741909-induced ROS generation, JNK activation, and apoptosis, whereas the ROS generation was not affected by other antioxidants, such as NAC, rotenone, L-NAME, DSE, naproxen, and oxypurinol. Interestingly, NDGA, aesculetin, baicalein, and caffeic acid are all reported to inhibit LOXs through their antioxidant activity. Nevertheless, those antioxidants mediated antagonist effect could be LOX independent because LOX inhibitors MK886 and zileuton, which do not have any intrinsic antioxidant activity, were not effective in blocking the NSC-741909-mediated ROS generation, nor did LOX specific siRNAs block NSC-741909-induced ROS generation and cell killing (Additional file 2). In addition, NAC, which acts as a precursor of GSH synthesis, did not attenuate the NSC-741909-mediated ROS generation, which suggests that the cellular reduction and oxidation regulated by intracellular GSH may not be very important for the NSC-741909-induced ROS production and cell death effects.

## Conclusion

Taken together, our results demonstrate that NSC-741909-induced apoptosis in human lung cancer cells is mediated by the generation of ROS. Blocking the formation of ROS could sufficiently inhibit the effects of NSC-741909, including JNK activation, cell growth suppression, and apoptosis. These results indicate that the oxidative stress-mediated sustained activation of JNK and subsequent induction of apoptosis is likely the primary mechanism by which NSC-741909 exerts its antitumor cell activity.

## Competing interests

The authors declare that they have no competing interests.

## Authors' contributions

XW and WG carried out experiments and prepared the manuscript. SW designed the synthesis route of the compound. LW, PH and JL participated in the cell culture and cell viability test. BF provided initial conception and supervised the project. All authors read and approved the final manuscript.

## Supplementary Material

Additional file 1 Chemical Structures of oncrasin-1 and NSC-741909. Click here for file

Additional file 2 **NSC-741909-induced apoptosis and ROS in the presence or absence of siRNA of 5-, 12-, and 15-Lox**. Control siRNA and 5-, 12- and 15-Lox siRNA were obtained from Dharmacon (Chicago, IL, USA). siRNA transfection were performed as we previously reported (Wei X, et al., J Biol Chem 2009, **284**:16948-16955). H460 cells were treated with PBS or transfected with various siRNA for 24 h, and then treated with 1 μM for another 24 h. Apoptosis and ROS analysis were performed as described in the manuscript. A) Cell cycle analysis. The number in each panel represents apoptotic cells (%). B) ROS levels. Click here for file

Additional file 3 **IC50s and levels of MKPs of lung cancer cell lines described in this manuscript and in NCI's 60 cell line panel**. The MKPs levels were obtained from microarray data provided by Dr. John Minna (University of Texas Southwestern Medical School, Dallas, USA) or obtained from NCI's Molecular Targets website http://dtp.nci.nih.gov/mtweb/search.jsp. Click here for file
