# Supplementary figures and images for: Oxidative stress in NSC-741909-induced apoptosis of cancer cells
Source: J Transl Med. 2010 Apr 16;8:37. doi: 10.1186/1479-5876-8-37 (PMC2873373; doi:10.1186/1479-5876-8-37)

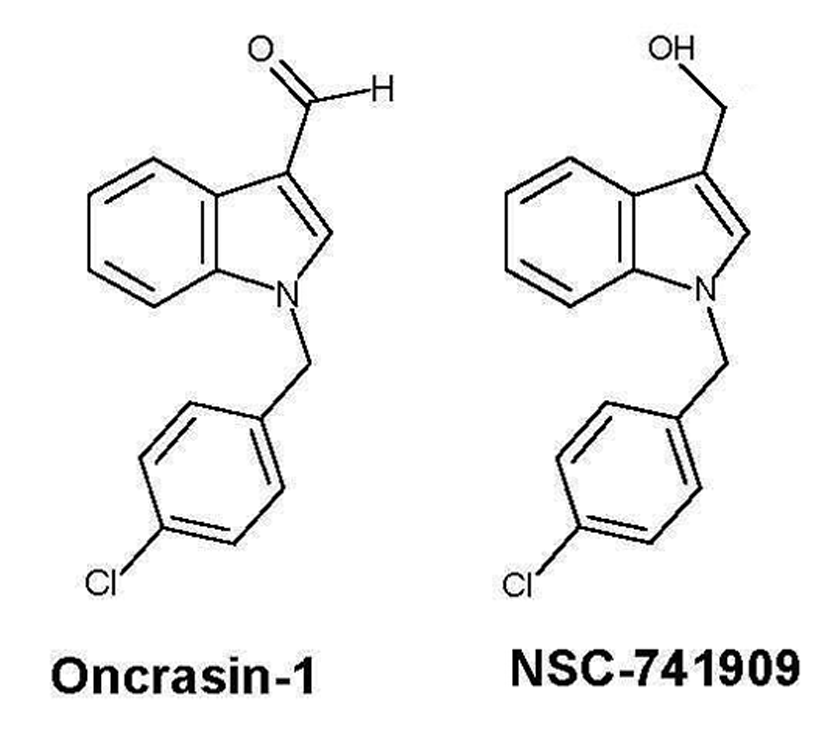

Supplement: Additional file 1 — Chemical Structures of oncrasin-1 and NSC-741909. [file 1479-5876-8-37-S1.TIFF]

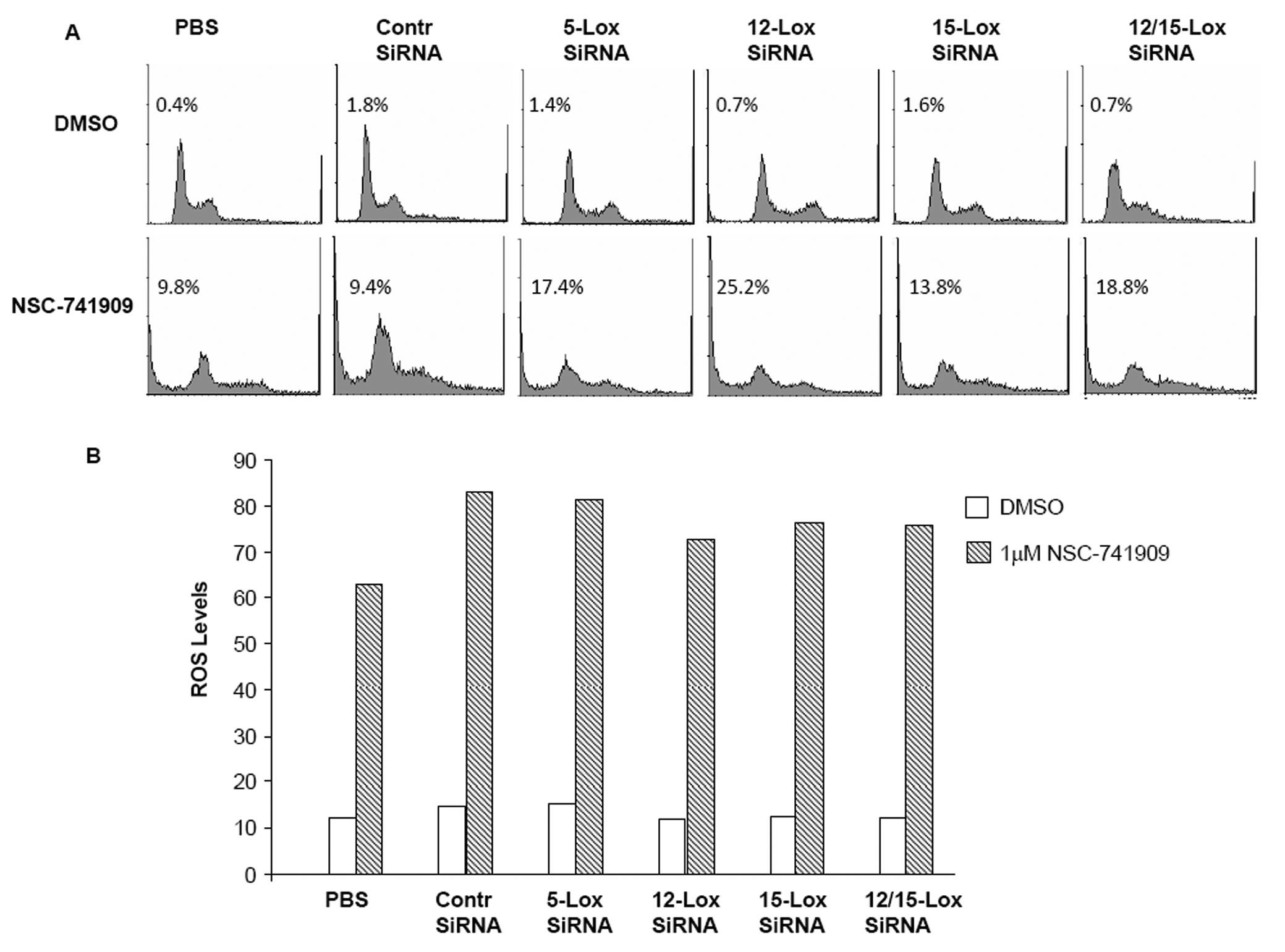

Supplement: Additional file 2 — NSC-741909-induced apoptosis and ROS in the presence or absence of siRNA of 5-, 12-, and 15-Lox. Control siRNA and 5-, 12- and 15-Lox siRNA were obtained from Dharmacon (Chicago, IL, USA). siRNA transfection were performed as we previously reported (Wei X, et al., J Biol Chem 2009, 284:16948-16955). H460 cells were treated with PBS or transfected with various siRNA for 24 h, and then treated with 1 μM for another 24 h. Apoptosis and ROS analysis were performed as described in the manuscript. A) Cell cycle analysis. The number in each panel represents apoptotic cells (%). B) ROS levels. [file 1479-5876-8-37-S2.TIFF]

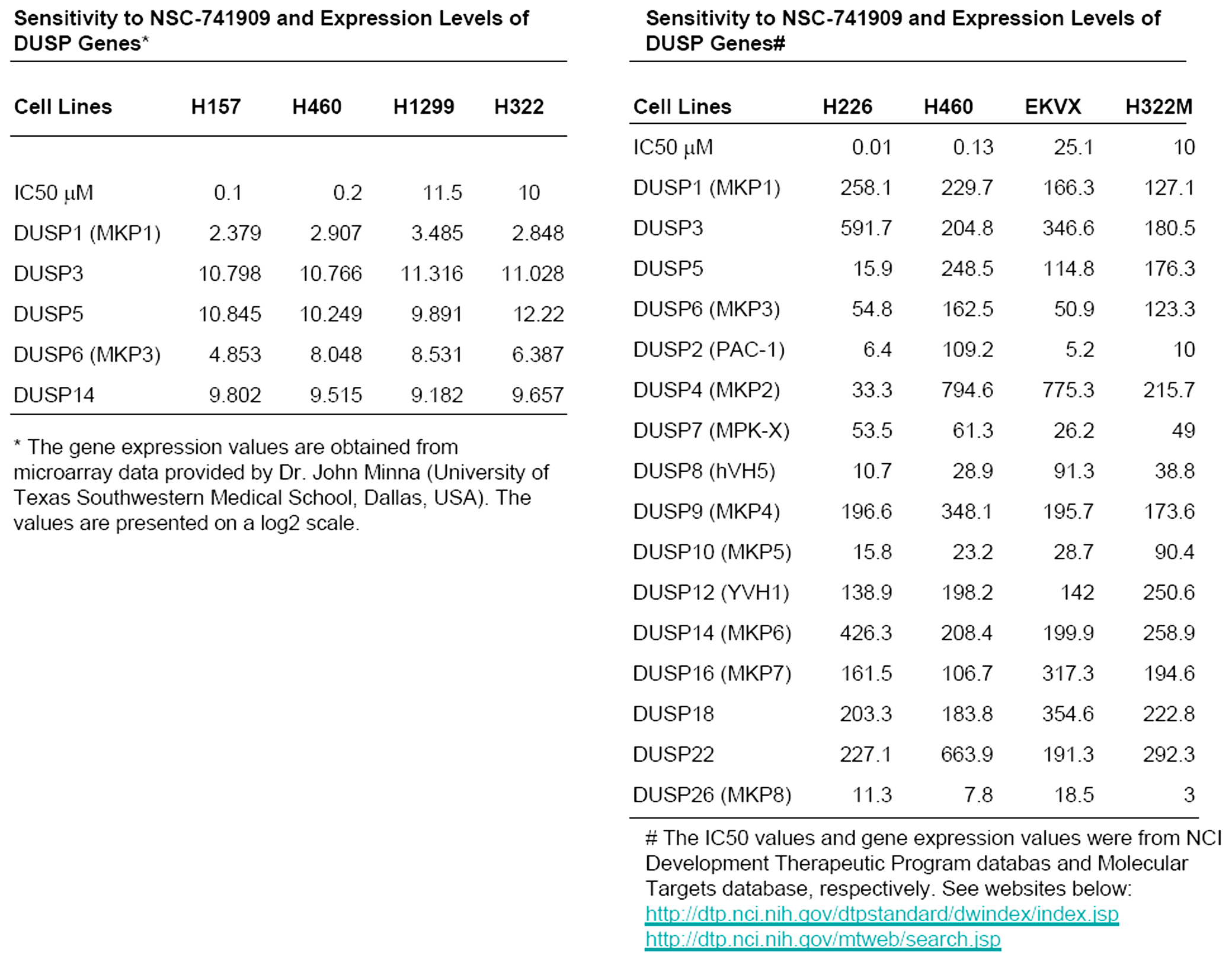

Supplement: Additional file 3 — IC50s and levels of MKPs of lung cancer cell lines described in this manuscript and in NCI's 60 cell line panel. The MKPs levels were obtained from microarray data provided by Dr. John Minna (University of Texas Southwestern Medical School, Dallas, USA) or obtained from NCI's Molecular Targets website http://dtp.nci.nih.gov/mtweb/search.jsp. [file 1479-5876-8-37-S3.TIFF]
